# Potential protective effects of bilirubin following the treatment of neonatal hypoxic-ischemic encephalopathy with hypothermia therapy

**DOI:** 10.1042/BSR20182332

**Published:** 2019-06-04

**Authors:** Liangyan Zou, Hao Yuan, Qing Liu, Chunmei Lu, Laishuan Wang

**Affiliations:** Department of Neonatology, Children’s Hospital of Fudan University, Shanghai 201102, China

**Keywords:** Bayley Scales, bilirubin, hypoxic-ischemic encephalopathy, Therapeutic hypothermia

## Abstract

**Background**: Therapeutic hypothermia (TH) is the standard therapy for hypoxic-ischemic encephalopathy (HIE) and is associated with a wide range of physiological changes. **Objective**: We re-evaluated the effects of HIE and TH on bilirubin measurements following HIE in a center involved in the China cooling randomized controlled trial (RCT). **Methods**: Serial serum bilirubin concentrations measured during the first week of life were compared among the HIE + NT (normothermia) group, HIE + TH treatment group and control group (without HIE). Survivors of HIE were followed and assessed at approximately 2 years of age, and the results were correlated with peak bilirubin levels during the first week of life. **Results**: One hundred and thirty-eight infants were available for analysis. Significantly lower bilirubin levels were recorded in the HIE + NT group than in the controls (*P*<0.05). Significant differences were not observed among the patients in the HIE + NT group (mild to severe) or between the HIE + TH group and the HIE + NT group at any time point (*P*>0.05). The peak serum bilirubin concentrations recorded at 96 h of age showed a good correlation with the results of the Bayley Scales of Infant and Toddler Development, third edition (BSID-III) (*P*=0.02). **Conclusion**: Bilirubin potentially exerts a neuroprotective effect during the first week of life, and low temperature does not affect the possible antioxidant function of bilirubin during TH following HIE.

## Introduction

Despite treatment with therapeutic hypothermia (TH) for neonatal hypoxic-ischemic encephalopathy (HIE), approximately 50% of treated newborns have disabilities at 12–18 months of age [1,2]. In addition, HIE is often associated with multiorgan dysfunction, and as the standard therapy for HIE, TH is associated with a wide range of physiological changes that affect almost every organ system and function. An awareness of these effects is essential for optimum management of these critical patients [3,4].

HIE can coincide with hyperbilirubinemia during the first few days (similar time window) after birth [5], and bilirubin, the end product of heme catabolism in mammals, is generally regarded as a potentially neurotoxic waste product that must be promptly excreted [6].

However, accumulating evidence indicates that early physiological increases in serum bilirubin levels in neonates may provide a protective antioxidant defense mechanism to compensate for otherwise deficient antioxidant enzymes, particularly in neonates with increased susceptibility to ischemia/reperfusion injury, such as infants with HIE [7]. In our clinical practice, patients with HIE were less likely to need or did not need phototherapy, and we speculate that bilirubin metabolism may be affected by HIE. We tested this potential hypothesis by examining the relationship between serum bilirubin concentrations and HIE severity, as well as the correlation between the serum bilirubin content and long-term neurodevelopmental outcomes.

Until recently, no studies had investigated the relationship between TH and bilirubin levels. A small-scale retrospective observational study reported significantly lower bilirubin concentrations than expected during the first four postnatal days in asphyxiated infants with HIE who were treated with TH [7].

After a multicenter randomized controlled trial (RCT) was completed in 2005 (NCT00890409) and before we universally adopted cooling as a standard therapy for HIE in 2010, we treated infants with HIE based on the approach from China’s selective head cooling multicenter RCT [8].

Thus, we investigated the relationship between the bilirubin concentration and low temperature in a retrospective review of infants with HIE who were treated with TH. We sought to verify whether hyperbilirubinemia was a risk factor for HIE and whether hypothermia affected bilirubin levels, and tentatively investigated the relationship between bilirubin levels and long-term neurodevelopmental outcomes.

## Methods

### Study design

This prospective RCT study was conducted to evaluate a possible correlation between bilirubin levels and the severity of HIE (mild to severe) in neonates treated with or without TH at our tertiary university hospital (Children’s Hospital of Fudan University, CHOF) over a period of 8 years (2003–2010). The study protocol was approved by the Research Ethics Committee of CHOF and written informed consent was obtained from the neonates’ parents before enrollment. Our study has been carried out in accordance with the World Medical Association Declaration of Helsinki.

Infants were eligible if they had a gestational age (GA) of 37 weeks, a birth weight (BW) of 2500 g, and were admitted to the neonatal intensive care unit (NICU) within 6 h of birth due to clinical evidence of perinatal hypoxia-ischemia or a diagnosis of encephalopathy. The inclusion criteria and the exclusion criteria were the same as our RCT study [8] and were omitted here. The randomization codes were generated by a computer and supplied in numbered, sealed, opaque envelopes.

### Procedure for selective head cooling

A semiconductor-controlled water circulation cooling device (YJW608-04B; Henyang Radio Manufactory, Hunan, China) was used for head cooling. Head cooling was initiated within 6 h of birth and continued for 72 h, followed by spontaneous rewarming [8].

Infants in the control group were cared for in radiant warmers to maintain a rectal temperature between 36 and 37.5°C. Other than the different temperature management protocols, infants in both groups received the same clinical care, monitoring of their vital signs and surveillance for organ dysfunction. We retrospectively selected another 49 patients without HIE as normal controls, and bilirubin measurements for all of these patients were available in the first week of life. Among the normal controls, individuals with Coombs-positive hemolytic anemia and/or a glucose-6-phosphate dehydrogenase (G-6-PD) deficiency were excluded.

### Monitoring of adverse events

Clinical demographic data, including Apgar scores, GA, BW, seizures, and treatment with phenobarbital, were also collected according to the protocol. In addition, we also routinely monitored severe arrhythmias, refractory hypotension, moderate or severe scleroderma, severe bleeding, liver dysfunction, and thrombocytopenia according to the protocol.

### Bilirubin measurements, MRI scans and scores

Total serum bilirubin (TSB) measurements were recorded as close as possible to 6, 12, 24, 48, 72, 96, 120, 132, 144, 156 and 168 h of age for each infant. Blood samples (50 µl) were collected to routinely measure bilirubin levels in our clinical biochemistry laboratory using a spectrophotometer or from a bedside blood gas analysis. Samples were collected beginning on day 1 of life and daily thereafter as long as an indwelling catheter was in place (5–7 days); in a few instances, transcutaneous bilirubin (TCB) results were used instead. The means and 95% confidence intervals were calculated for each defined time within each group during the first 7 days of life. These values were plotted for infants with varying severities of HIE and for controls as well. Clinical MRI scans were routinely performed on either a Siemens 1.5-T Avanto or 3.0-T Trio (Siemens Medical, Erlangen, Germany) scanner according to our MRI protocol for infants with HIE, and the results were assessed using the MRI scoring system developed by Barkovich et al. [9]. More extensive injuries resulted in higher scores.

### Long-term follow-up

All infants with HIE who underwent TH or NT and survived were routinely tested at 18–24 months of age using the Bayley Scales of Infant and Toddler Development, third edition (BSID-III) as [10]. Cognitive and motor composite scores were determined. The standardized norm for each of the scales is 100, with an SD of 15. BSID-III composite scores ≥ 85 were classified as normal neurodevelopmental outcomes, and composite scores < 85 were classified as abnormal outcomes. Additionally, the correlation between the measured peak serum bilirubin level and the BSID-III score was also determined separately.

### Statistical analysis

Normally distributed data are presented as means ± SD. Between-group comparisons of continuous variables were conducted with Student’s *t* test. Proportions are presented as percentages, and between-group comparisons were conducted with the χ^2^ test or Fisher’s exact test. We also performed linear regression analyses using Pearson’s correlation test to determine whether the peak bilirubin concentration predicted the BSID-III composite score. Statistical analyses were performed using SPSS version 22 (SPSS, Inc., Chicago, Illinois), and a value of *P*<0.05 was considered statistically significant.

## Results

During the 8-year study period, 45 infants with HIE were randomized to the HIE + TH group and 44 infants with HIE were randomized to the HIE + NT group. Of the 59 potential normal control infants without HIE (control group), 10 were admitted because of pathological hyperbilirubinemia with hemolysis and were excluded from further analyses, leaving 49 normal control infants. Of these infants, 28 were admitted with pneumonia, 12 with gastrointestinal problems, 3 with cardiac problems, 3 with infections, and 3 with other hematological (non-hyperbilirubinemic) problems.

The characteristics of the infants with HIE who were included in this prospective study are presented in Table 1. Statistically significant differences in baseline characteristics were not observed. The nasopharyngeal temperature was maintained at approximately 34°C in the HIE + TH group. In the HIE + NT group, the average nasopharyngeal and rectal temperatures were stable and were maintained between approximately 36 and 36.5°C.

**Table 1:** Baseline clinical characteristics of the HIE + TH and HIE + NT groups

	HIE+TH (*n*=45)	HIE+NT (*n*=44)	*P*
Mode of delivery			
Cesarean section (C/S)	18 (40%)	17 (38%)	0.11
Assisted	13 (29%)	13 (29%)	0.90
Spontaneous	14 (31%)	14 (31%)	0.91
GA (weeks)	38.5 ± 1.0	38.6 ± 1.2	0.19
BW (g)	3150 ± 284	3141 ± 302	0.23
Male sex	31 (69%)	29 (65%)	0.17
Fetal distress	14 (31%)	15 (34%)	0.18
Complications of pregnancy	35 (78%)	34 (78%)	0.91
Assisted ventilation	15 (33%)	13 (30%)	0.08
Seizures	16 (35%)	15 (33%)	0.13
Treated with phenobarbital	15 (34%)	15 (34%)	0.12
Severity of HIE			
Mild	8 (17%)	7 (15%)	0.32
Moderate	29 (64%)	28 (62%)	0.16
Severe	8 (17%)	9 (20%)	0.52
5-min Apgar score ≤ 5	42 (95%)	41 (93%)	0.45
Onset of therapy (h after birth)	4.0 ± 1.2	3.9 ± 1.1	0.24
MRI performed (*n*, %)	42 (93%)	42 (95%)	0.87
BSID-III performed (*n*, %)	38 (84%)	36 (82%)	0.53

Values are presented as means ± SD, and between-group comparisons of continuous variables were conducted with Student’s *t* test. The *chi* square test or Fisher’s exact test was applied to calculate the ‘*P*’ value. A ‘*P*’ value of <0.05 was considered statistically significant.

Approximately one-third of infants with HIE developed seizure episodes during the course of hospitalization. All infants experiencing seizures were treated with phenobarbital; no difference was observed in the occurrence of seizures between the HIE + TH and HIE + NT group (35 versus 34%, Table 1). The serum bilirubin concentrations of the phenobarbital-treated infants were not different from the other infants with HIE at any time point (unpublished data).

The distribution of HIE severity, Apgar scores and onset of therapy (TH or NT), as well as MRI scans and BSID-III assessments were all comparable between the HIE + TH and HIE + NT groups. Death (time and cause) and major adverse events were similar between the two groups (Table 2), suggesting a lack of significant adverse effects of TH on neonatal HIE.

**Table 2:** Comparisons of major outcomes between the HIE + TH and HIE + NT groups

	HIE+TH (*n*=45)	HIE+NT (*n*=44)	*P*
Death (*n*, %)	6 (13%)	6 (14%)	0.43
Death ≤ 3 days	4 (9%)	4 (9%)	
Death > 3 days	2 (4%)	2 (4.5%)	
Cause of death			
HIE	1 (2.2%)	2(4.5%)	
Respiratory failure	2 (4.4%)	2 (4.5%)	
Kidney failure	1 (2.2%)	0	
Others	2 (4.4%)	2 (4.5%)	
Raised liver enzymes	10 (22%)	9 (20%)	0.63
Platelet count < 100000 per ml	6 (13.3%)	7 (15.9%)	0.15
Moderate or severe scleroderma	1 (2.2%)	0	
Severe bleeding	3 (6.6%)	2 (4.5%)	0.08
Oliguria	4 (8.8%)	4 (9.0%)	0.23
Refractory hypotension	3 (6.6%)	4 (9.0%)	0.09
Severe arrhythmia	2 (4.4%)	2 (4.5%)	0.18
MRI scores	2 (0–4)	3 (1–5)	0.04
BSID-III (*n*)	38	36	
Normal	28 (74%)	16 (44%)	<0.01
Abnormal	10 (26%)	20 (56%)	<0.01
Motor composite score	108 ± 11	85 ± 15	<0.02
Cognitive composite score	99 ± 10	101 ± 12	0.98

Values are presented as means ± SD, and between-group comparisons of continuous variables were conducted with Student’s *t* test. The *chi* square test or Fisher’s exact test was applied to calculate the ‘*P*’ value. A ‘*P*’ value of <0.05 was considered statistically significant.

A brain MRI was performed at a median postnatal age of 7 days. MRI data were not available for five infants (5.6%) who died before an MRI could be performed (three in the HIE + TH group and two in the HIE + NT group). Although the sample size was small, the MRI scores were statistically significantly different between the HIE + TH and HIE + NT groups (*P*=0.04), suggesting a protective effect of TH from the neuroimaging perspective. Characteristics of hyperbilirubinemia-induced brain injury were not observed on any of the available MRI scans.

The BSID-III was administered to 74 children (38 in the HIE + TH group and 36 in the HIE + NT group) at a median age of 21 months (IQR 18–27), and 15 infants died or were lost to follow-up (6 deaths and 1 lost to follow-up in the HIE + TH group and 6 deaths and 2 lost to follow-up in the HIE + NT group). Twenty-eight survivors in the HIE + TH group (74%) had a normal BSID-III outcome, whereas only 16 (44%) subjects in the HIE + NT group had a normal outcome at 18–24 months of age, resulting in a significant difference (*P*<0.01) (Table 2).

The BSID-III cognitive composite score was comparable between the two groups (99 versus 101, *P*=0.98), while a higher motor composite score was observed in the HIE + TH group than in the HIE + NT group (108 versus 85, *P*<0.02) (Table 2).

**Figure 1:**
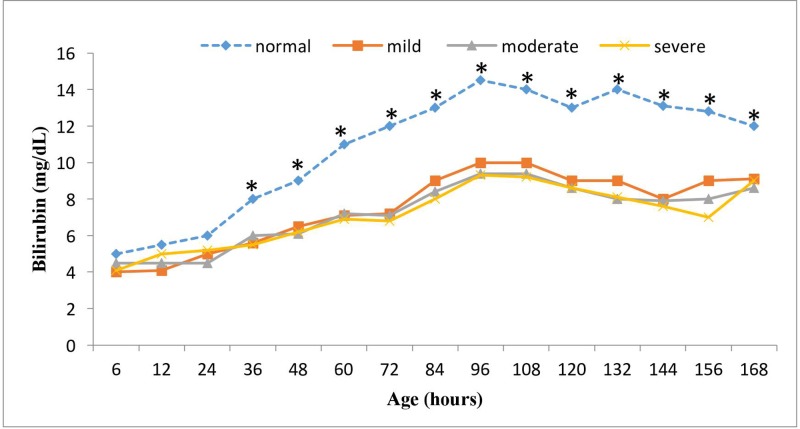
TSB concentrations over time during the first 7 days of life Mean serum bilirubin concentrations were plotted for neonates in the HIE + NT group [mild, *n*=7, moderate, *n*=30, and severe, *n*=7], and normal age-matched controls (*n*=49) over time. The HIE + NT group presented significantly lower TSB concentrations than the control group at each time point assessed (*) (*P*<0.05), except during the first 24 h. No differences were observed among the mild, moderate and severe HIE + NT groups (*P*>0.05).

The mean TSB concentrations in infants with any stage of HIE + NT groups were significantly lower than the controls at each time point from 24 to 168 h after birth (*P*<0.05, Figure 1). By 96 h of age, when the peak level of bilirubin occurs, the bilirubin concentration was 9.6 (8.4, 10.8) mg/dl in the mild HIE + NT group versus 14.5 (12.5, 16.5) mg/dl in the control group (*P*=0.02). The figure also shows the standard serum bilirubin concentrations during the first 7 days of life in control infants, which are similar to the Buhtani standard curve [9]. Notably, 100% of infants in the asphyxiated group (TH and NT) remained below the 60th percentile of these curves (Figure 1).

The bilirubin levels in the various stages of HIE + HT groups were not significantly different (*P*>0.05) from the HIE + NT groups. Two infants in the HIE + NT group had mild Coombs-positive (ABO hemolytic) anemia (11 g/dl) and two infants in the HIE + TH group were diagnosed with a G-6-PD deficiency; however, their peak bilirubin levels never exceeded 13 mg/dl.

**Figure 2:**
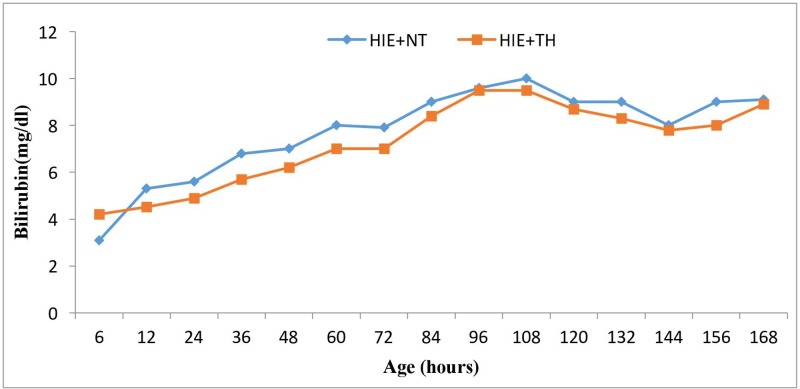
Mean TSB concentrations over time during the first 7 days of life in the HIE + NT group (*n*=38) and HIE + TH group (*n*=39) Bilirubin levels are independent of whether the babies underwent TH or not (*P*>0.05).

Furthermore, the mean bilirubin level in neonates in the HIE + NT group during the first 7 days of life was 7.7 mg/dl, while it was 7.2 mg/dl in neonates in the HIE + TH group; this difference was not statistically significant (*P*>0.05) (Figure 2). The daily mean bilirubin levels were very similar in infants in the HIE + NT group and infants in the HIE + TH group (*P*>0.05) (Figure 2).

**Figure 3:**
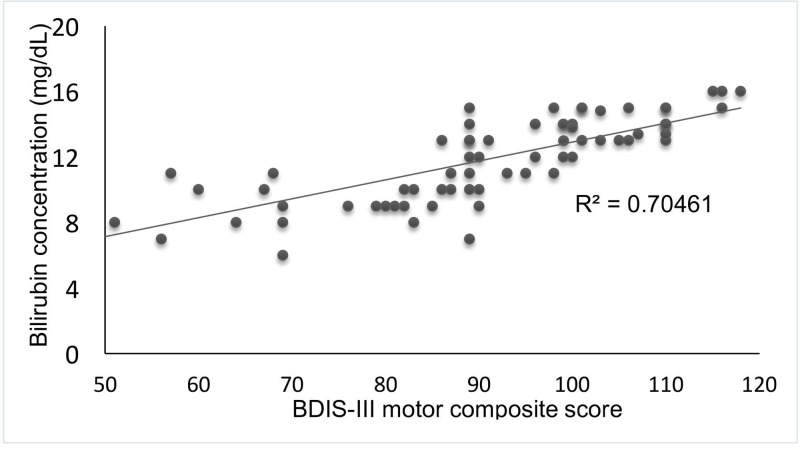
Analysis of the correlation between the peak TSB concentration and the BSID-III score The peak TSB concentrations (mg/dl) (96 h of age) are plotted against the BSID-III motor composite scores at 18–24 months of age for infants with HIE. A significant correlation was noted (*R²* = 0.7046; *P*=0.02).

The peak serum bilirubin level and BSID-III motor composite score were strongly correlated in all infants with HIE and were not affected by TH (*R²* = 0.7046; *P*=0.02) (Figure 3).

## Discussion

Normal bilirubin metabolism is complex and multifaceted following perinatal asphyxia and treatment with TH for HIE [3,12].

Asphyxiated infants had lower bilirubin concentrations in the first few days after birth than non-asphyxiated control infants who were admitted to our NICU during the same period or infants included in previously published studies [8]. The mechanism underlying this change is unclear and debatable, but bilirubin is now accepted to be one of the body’s natural antioxidants, contributing to 10–30% of the total antioxidant capacity in newborns [12–14]. Therefore, bilirubin might play a potential role and be consumed as an antioxidant during severe neonatal oxidative stress, such as perinatal asphyxia.

In fact, the antioxidant effects of bilirubin have been extensively explored in neonatal diseases, such as retinopathy of prematurity (ROP), necrotizing enterocolitis (NEC) and kernicterus [15–21]. However, in terms of asphyxia, more attention has been paid to its possible neurotoxic effects than to its neuroprotective effects. Furthermore, the effects of bilirubin levels depend on the magnitude of the change: the highest levels (greater than the exchange transfusion level) may exert deleterious effects. Nevertheless, because none of the infants in the HIE + TH and HIE + NT groups required phototherapy for hyperbilirubinemia, at the physiological jaundice level, higher hyperbilirubinemia may be safe or even protective for infants with HIE, which was verified by the positive correlation between the peak bilirubin level and BSID-III motor composite score.

The brain is the organ that is most susceptible to oxidative injury because of its rapid metabolic and oxygen consumption rates, its high content of easily oxidized membrane lipids, and its limited antioxidant mechanisms [19]. Our data did not enable us to directly distinguish between the effects of asphyxia on bilirubin consumption and the possible effects of cooling on bilirubin production, in contrast with previous similar but small-sized retrospective observational studies [5,7].

In human infants, serum bilirubin levels significantly correlate (*P*<0.005) with the total antioxidant status *in vivo*, as measured by the peroxyl radical trapping capability of human blood [14]. According to several studies [22–26], infants suffering from conditions that are possibly associated with oxygen radical-mediated mechanisms have lower bilirubin concentrations than infants without proposed oxygen radical involvement. Therefore, we speculate that following HIE, bilirubin may be consumed due to its antioxidant potential.

We did not identify any significant effect of TH on bilirubin metabolism. We do not have any explanation for this finding. As shown in the study by Dani et al. [5], HIE and hypothermia may independently decrease TSB levels, and changes in TSB concentrations in infants with HIE may be due to the hypoxia-mediated repression of heme-oxygenase (HO) expression and represent a defensive strategy for limiting brain injuries in these patients.

In addition, animal studies have supported a protective effect of hyperbilirubinemia *in vivo*; it is capable of protecting against hyperoxic damage and exerts neuroprotective effects on focal ischemia [20]. TH significantly attenuates the bilirubin-induced alterations in brain cell membrane function and energy metabolism in newborn piglets and decreases neuronal cell death induced by bilirubin toxicity [26]. We also identified a correlation between the BSID-III motor composite score and peak bilirubin level, which further characterizes the possible neuroprotective effects of bilirubin following HIE.

Thus, our findings may be clinically relevant because they suggest the possible existence of mechanisms that prevent infants who suffered asphyxia during the perinatal period from subsequently developing neonatal hyperbilirubinemia that might increase the risks of cerebral damage and encephalopathy [5].

The present study has several limitations. First, we only included data from one center with a relatively small sample size. Due to the relatively small number of patients, we combined all infants with HIE (mild, moderate and severe) in a group to investigate the effects of hypothermia, which made it difficult to determine the effect of the severity of HIE. Second, our study offers only indirect evidence that bilirubin is consumed *in vivo*. However, the low rate of increase in serum bilirubin levels is not well explained by classic bilirubin physiology alone. Third, as the present study was performed at a single center, generalizability to other centers might be limited. However, the follow-up rate was 80%, making it a reliable representation. Fourth, different clinical etiologies of hyperbilirubinemia may have exhibited pathophysiological relationships with the outcomes. The small sample size prevented subgroup analyses. Finally, although the present study employed a prospective RCT design, direct measures of antioxidant concentrations, bilirubin production or consumption were not performed; thus, our suggestions for the pathophysiological mechanisms are only speculations.

In addition, our age-matched control infants were not actually healthy infants. In this context, we were fortunate to have the Bhutani nomogram [12], which provides population-wide norms, for further comparison, as an additional control that offers a certain advantage because it has been recognized and included in the American Academy of Pediatrics (AAP) hyperbilirubinemia guidelines.

None of the infants in the study had bilirubin levels near the 60th percentile and no infants required phototherapy, supporting the observation that HIE infants are clinically distinct from the normal, term neonatal population in terms of their bilirubin levels.

In summary, in our prospective RCT study, bilirubin potentially exerts neuroprotective effects during TH following HIE.

## Abbreviations

BSID-III: Bayley Scales of Infant and Toddler Development, third edition
BW: birth weight
CHOF: Children’s Hospital of Fudan University
GA: gestational age
G-6-PD: glucose-6-phosphate dehydrogenase
HIE: hypoxic-ischemic encephalopathy
NICU: neonatal intensive care unit
RCT: randomized controlled trial
TH: therapeutic hypothermia
TSB: total serum bilirubin
